# Directed Evolution of Human Heavy Chain Variable Domain (V_H_) Using *In Vivo* Protein Fitness Filter

**DOI:** 10.1371/journal.pone.0098178

**Published:** 2014-06-03

**Authors:** Dong-Sik Kim, Hyung-Nam Song, Hyo Jung Nam, Sung-Geun Kim, Young-Seoub Park, Jae-Chan Park, Eui-Jeon Woo, Hyung-Kwon Lim

**Affiliations:** 1 Antibody Engineering, Mogam Biotechnology Research Institute, Yongin, Republic of Korea; 2 BioMedical Proteomics Research Center, Korea Research Institute of Bioscience and Biotechnology, Daejeon, Republic of Korea; 3 Department of Biotechnology and Bioinformatics, Korea University, Sejong, Republic of Korea; Russian Academy of Sciences, Institute for Biological Instrumentation, Russian Federation

## Abstract

Human immunoglobulin heavy chain variable domains (V_H_) are promising scaffolds for antigen binding. However, V_H_ is an unstable and aggregation-prone protein, hindering its use for therapeutic purposes. To evolve the V_H_ domain, we performed *in vivo* protein solubility selection that linked antibiotic resistance to the protein folding quality control mechanism of the twin-arginine translocation pathway of *E. coli*. After screening a human germ-line V_H_ library, 95% of the V_H_ proteins obtained were identified as V_H_3 family members; one V_H_ protein, MG2x1, stood out among separate clones expressing individual V_H_ variants. With further screening of combinatorial framework mutation library of MG2x1, we found a consistent bias toward substitution with tryptophan at the position of 50 and 58 in V_H_. Comparison of the crystal structures of the V_H_ variants revealed that those substitutions with bulky side chain amino acids filled the cavity in the V_H_ interface between heavy and light chains of the Fab arrangement along with the increased number of hydrogen bonds, decreased solvation energy, and increased negative charge. Accordingly, the engineered V_H_ acquires an increased level of thermodynamic stability, reversible folding, and soluble expression. The library built with the V_H_ variant as a scaffold was qualified as most of V_H_ clones selected randomly were expressed as soluble form in *E. coli* regardless length of the combinatorial CDR. Furthermore, a non-aggregation feature of the selected V_H_ conferred a free of humoral response in mice, even when administered together with adjuvant. As a result, this selection provides an alternative directed evolution pathway for unstable proteins, which are distinct from conventional methods based on the phage display.

## Introduction

The variable domain of heavy or light chain (V_H_ or V_L_) of a human immunoglobulin G (IgG) molecule is the smallest part of the antibody that preserves the original binding activity. Although variable domains have short serum half-lives and lack effector function, their format flexibility by adopting immune cell engaging strategy or introducing a long-acting module can ameliorate these defects [1]–[3]. Furthermore, their ability to access occluded or hidden epitopes, superior bio-distribution, and cost-effective production make variable domains potentially useful in therapeutic applications for which full IgG molecules are not appropriate [4]–[6].

When not assembled with each other, instability problem of V_H_ and V_L_ of human IgG is a major concern for biotechnological applications since Ward et al. reported that such V_H_ domains are relatively sticky resulting in tendency to aggregate [7]. This aggregation is primarily due to interactions between hydrophobic patches residing at the interface between V_H_ and V_L_. Direct replacement of the interfacial hydrophobic residues of V_H_ or V_L_ with hydrophilic amino acids has been partially successful in improving protein stability. Three hydrophilic substitutions (G44E/L45R/W47G) improve the solubility of V_H_ [8]–[10], but these changes also decrease expression yield and thermal stability due to the resultant deformations of the β-sheet structure [11]–[13].

In addition to rational mutation strategies, several groups have adopted combinatorial approaches to engineer human V_H_ or V_L_. Jespers et al. screened a combinatorial CDR library bound to protein A for aggregation-resistant V_H_, using panning phage display under heat-denatured conditions [14]. They found that mutations in the CDRs of human V_H_ can increase solubility and promote reversible folding. Without the use of heat denaturation in phage display, Barthelemy et al. isolated various mutant V_H_ domains with an increased stability and solubility [15]. To eliminate the complicated step involving *in vitro* protein A panning, To et al. selected monomeric human V_H_ domains directly from bacterial lawns by plaque size [16]. These variant techniques notwithstanding, most screenings of engineered V_H_ domains have been conducted using phage display and protein A-binding activity.

On the other hand, *in vivo* genetic selection methods distinct from *in vitro* phage display have been applied in efforts to improve protein solubility [17], [18]. In one such *in vivo* method, the twin-arginine translocation (Tat) pathway was exploited as an *in vivo* protein fitness filter for fast folding and solubility of protein of interests including single chain Fv [19]–[21]. However, such approaches have not been attempted for V_H_ or V_L_ alone. In the current study, we applied this system to evolve human V_H_ toward greater stability and characterized the structural hallmarks to greater stability and solubility.

## Materials and Methods

### Ethics Statement

All animal experiments were performed in accordance with the guidelines for the care and use of laboratory animals recommended by the Ministry of Food and Drug Safety of Republic of Korea. The experimental procedures were approved by the Mogam Animal Care and Use Committee. Currently, Mogam Animal Care and Use Committee changed the name as the Green Cross Central Research laboratory Animal Care and Use Committee, by which the animal experiment closing report was reviewed and approved.

### Construction of the Tat-based genetic selection vector

**Figure 1:**
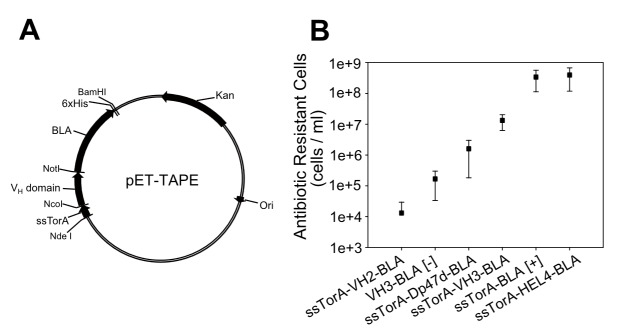
Verification of the selection system, TAPE. (A) Plasmid map of pET-TAPE. (B) Average number of ampicillin-resistant colonies from cultures harboring constructs for expression of a negative control (no Tat signal sequence, V_H_3-BLA [−]), positive control (Tat signal sequence and reporter gene only, ssTorA-BLA [+]), and published V_H_ domains (HuCal V_H_2, HuCal V_H_3, Dp47d, and HEL4). Each construct was expressed in LB medium containing 50 µg/ml ampicillin. Cultures were induced by the addition of 1 mM isopropylβD-1-thiogalactopyranoside for 3 h after inoculation. After the induction, cultures were spread onto agar plates containing 50 µg/ml ampicillin for colony counting. Data points are means and standard deviation for three independent experiments.

The vector system for screening of stable V_H_ domains was modified from the previous report [20], [22]. Briefly, TEM-1 β-lactamase (*BLA*) was ligated with the Tat signal sequence of trimethylamine N-oxide reductase (ssTorA) of *E. coli* in pET9a, yielding pET-TAPE (Figure 1A). Next, a fusion gene of ssTorA with the representative human immunoglobulin heavy chain variable domain V_H_ family type 2 (V_H_2) was synthesized (GenScript, USA). V_H_2 was used as a template for PCR using a 5′ primer (Table S1, primer 1) including an *Nde*I restriction site and a 3′ primer (Table S1, primer 2) including a *Not*I site, a 6×His tag, and a *BamH*I site, to yield the *Nde*I-ssTorA-V_H_2-*Not*I-6×His-*BamH*I gene. This gene was inserted between the *Nde*l and *BamH*I sites in the multi-cloning site of pET9a to yield pET9a-ssTorA-V_H_2. The *Not*I-*BLA*-*BamH*I segment was generated by PCR (Table S1: primer 3 as sense, primer 4 as antisense) using *BLA* as a template. This gene was inserted between the *Not*l and *BamH*I sites of pET9a-ssTorA-V_H_2, yielding pET9a-ssTorA-V_H_2-*BLA*, which was named pET-TAPE. A synthetic or human germ-line V_H_ library was constructed by replacing the V_H_2 gene in pET-TAPE.

### Library design and construction

cDNA for the human V_H_ library was obtained by reverse transcription of mRNAs from the liver, peripheral blood mononuclear cells, spleen, and thyroid (Clontech, Madison, WI, US) using various primers (Table S1: primers 5–12 as sense, and primers 13–15 as antisense). Each of cloned human V_H_ gene family (V_H_1, V_H_3, and V_H_5) was inserted between the *Nde*I and *BamH*I sites of pET-TAPE, yielding a pET-TAPE-V_H_ library with approximately 10^9^ distinct clones. Mutations were introduced by PCR using MG2x1 as the template and primers that introduced mutations at the first fragment (Table S1: primers 16 and 17) and the second fragment (Table S1: primers 18 and 19). Next, MG2x1 variant genes were synthesized by overlapping PCR of the two gene fragments using primers 16 and 19 (Table S1). After digestion of the MG2x1 variants with *Nco*I and *Not*I, the inserts were cloned into pET-TAPE, yielding the frame-mutation V_H_ library with approximately 10^8^ distinct clones.

### Setup for Tat-associated protein engineering (TAPE) system

**Figure 2:**
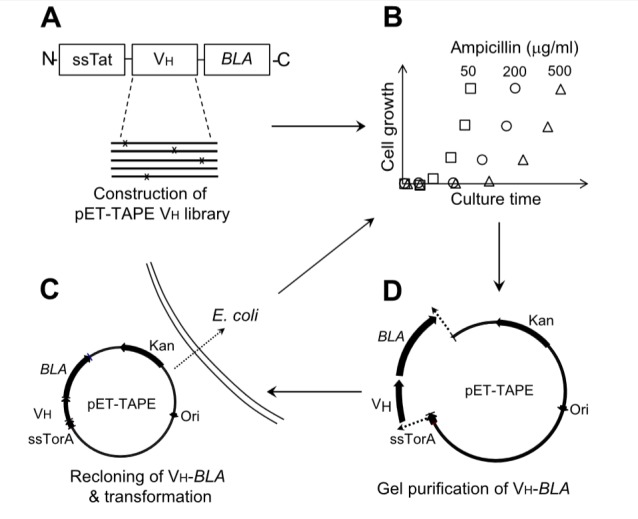
Schematic procedure for screening protein solubility using TAPE (‘liquid screen’). (A) Construction of the pET-TAPE V_H_ library (either germ-line or mutated) and transformation of the library into *E. coli*. (B) Liquid culture of the library with stepwise increases in antibiotic concentration. (C) Collection of plasmids and purification of the intact V_H_-BLA coding region. (D) Re-cloning of the V_H_-BLA gene into pET-TAPE between the *Nco*I and *BamH*I sites, and transformation into *E. coli*. Steps (B), (C), and (D) were repeated four times for each ampicillin concentration (50, 100, 250, and 500 µg/ml).

Along with the construction of pET-TAPE, the protocol implementing a liquid culture and rescuing correct size of gene of interests was conducted to screen protein solubility in high-throughput manner. The antibiotic resistance of *E. coli* is correlated to the translocation of soluble V_H_-BLA fusion protein into the periplasm via the Tat pathway. The TAPE system differs from previously described systems [20] in that soluble proteins are enriched in consecutive rounds of liquid culture with increasing concentrations of antibiotic. *E. coli* T7 Express LysY/I^q^ was transformed with the pET-TAPE-V_H_ library by electroporation. Transformants were cultured in SOC (20 g/l Bacto tryptone, 5 g/l Bacto yeast extract, 10 mM NaCl, 2.5 mM KCl, 10 mM MgCl_2_, 10 mM MgSO_4_, and 20 mM glucose) at 37°C for 1 h, and then inoculated and cultured in liquid LB media containing 50 µg/ml ampicillin. When OD (600 nm) reached 0.6, cells were collected by centrifugation and plasmid DNA was isolated. To prevent enrichment of false-positives in subsequent rounds of selection, isolated plasmids were restricted with *Nco*I and *BamH*I, and digests were subjected to gel electrophoresis to allow size selection of full-length V_H_-BLA genes. The size-selected V_H_-BLA genes were cloned between the *Nco*I and *BamH*I sites of pET-TAPE, and the resultant plasmids were transformed into *E. coli*. Subsequently, liquid culture was performed in repeated rounds with stepwise increases in the concentration of ampicillin up to 500 µg/ml. (Figure 2). After performing 3–5 consecutive cycles of liquid culture, clones were separated on an LB agar plate containing ampicillin and 50 µg/ml kanamycin.

### Host strains and plasmids

*E. coli* T7 Express LysY/I^q^ (New England BioLabs, MA, USA) was used as the host for the expression of the V_H_ domains and their fusion proteins. pET9a (New England BioLabs, MA, US) was used to construct the TAPE system, i.e., for expression of fusion proteins of various V_H_ domains and BLA. pET22b (New England Biolabs, MA, USA) was used to express the V_H_ domain alone. All other DNA manipulations were conducted according to common methods.

### Fractionation of soluble and insoluble V_H_

To determine the degree of soluble expression, individual V_H_ domains alone (i.e., without the BLA fusion) were expressed in *E. coli*. The soluble and insoluble fractions were separated after induction of V_H_ expression, followed by SDS-PAGE. Soluble and insoluble proteins were fractionated in lysis buffer (B-PER Reagent, Thermo Scientific, USA). The pellet was washed with PBS, and then resuspended in solubilization buffer (pH 7.4, 50 mM NaH_2_PO_4_, 6 M urea, 0.5 M NaCl, and 4 mM DTT) to obtain the insoluble fraction. Each fraction was prepared from the same quantity of cells to allow band intensities to be compared after gels were stained with Coomassie blue.

### Circular Dichroism

Purified V_H_ domains were diluted to 0.2 mg/ml. The purity of V_H_ domains used for CD measuremnt was demonstrated with SDS-PAGE (Figure S1). CD was measured using a spectropolarimeter (Jasco J-715 model, Jasco Inc, Easton, MD, US). T_m_ was defined as the temperature at which a 50% reduction in the soluble protein fraction was observed. The profile was recorded at a wavelength of 235 nm as the temperature gradually increased from 25 to 85°C at a rate of 1°C/min. All CD measurement were repeated 3 times for each V_H_ domain. The *p*-value (paired t-test) between two V_H_ domains was less than 0.005 for all possible pairs of the tested V_H_ domains.

### Recovery yield

The recovery yield was defined as the level of soluble V_H_ after heat denaturation. After aggregates were removed by centrifugation, the concentration of soluble V_H_ was determined according to the equation, c = A/ (E×b), where A is the absorbance at 280 nm, E is the molar extinction coefficient (M^−1^cm^−1^), b is the pathway length (cm), and c is the molar concentration (mol/l). The extinction coefficient was calculated using the amino acid composition, assuming that all pairs of cysteine residues were involved in disulfide bonds (web.expasy.org/protparam). Protein quality was confirmed by size-exclusion chromatography.

### Humoral immune response of mice to the screened V_H_

BALB/c mice (six per group) were intravenously injected with 10 µg MG2x1, MG8-14, or V_HH_ on 3 consecutive days. The injections were repeated at weeks 1, 4, and 8. Samples of immune sera were obtained every week, and mice were sacrificed at day 65. For intramuscular and subcutaneous injections, BALB/c mice (six per group) were injected with 1 or 10 µg of MG8-14 or V_HH_. V_HH_ is identical to V_HH_ #3E, which binds to tumor necrosis factor-α [23]. The injection was repeated every 2 weeks with a total of five injections. The mice were sacrificed 2 weeks after the final injection. Samples of immune sera were obtained every 2 weeks, 1 day before the next injection. To measure antibody titers, enzyme-linked immunosorbent assays were performed using 96-well plates coated with MG2x1, MG8-14, or V_HH_, and HRP-labeled goat anti-mouse antibody as a secondary antibody, followed by the addition of 3,3′,5,5′-tetramethylbenzidine and measurement of OD (490 nm).

## Results

### Verifying TAPE

**Figure 3:**
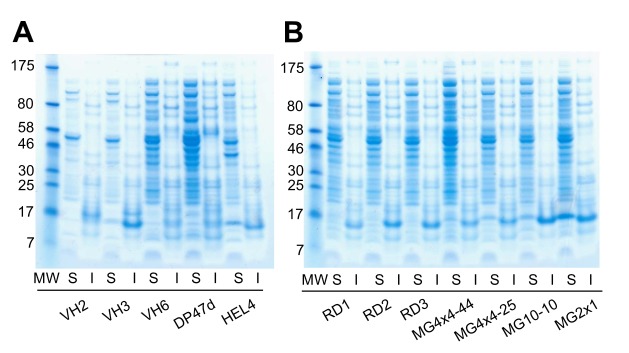
SDS-PAGE of soluble and insoluble fractions of *E.x coli*. (A) Previously characterized V_H_ domains (V_H_2, V_H_3, V_H_6, DP47d, and HEL4). (B) V_H_ domains chosen randomly from the human V_H_ germ-line library (RD1–3) or selected from the human germ-line library using TAPE (MG4x4-44, MG4x4-25, MG10-10, and MG2x1). Cultures expressing each V_H_ domain were harvested after induction at 25°C for 3.5 h, and soluble (S) and insoluble (I) fractions were prepared. Lane ‘MW’ contains a protein size marker; the size of each marker is indicated (in kD) to the left of each panel. In both panels, the mobilities of V_H_ domains correspond to the 15-kD protein size marker. Different parts from separating gels are grouped to align expression patterns for soluble and insoluble fraction of each V_H_ domain.

To verify whether TAPE system can discriminate between proteins of different solubilities, we applied this system to various published V_H_ domains whose soluble expression levels are well known. The V_H_ domains were cloned into the pET-TAPE vector (Figure 1A), allowing them to be expressed in *E. coli* as fusions with BLA and the Tat signal sequence of ssTorA. The antibiotic resistance of strains carrying each construct was measured by counting the cell number in cultures containing 50 µg/ml ampicillin. Cells expressing BLA alone (ssTorA-BLA [+], positive control) exhibited the highest resistance, and cells expressing HEL4 were approximately as resistant as the positive control (Figure 1B) [22]. Cells expressing the other representative V_H_3 family genes, Dp47d and V_H_3 (HuCAL), exhibited resistances intermediate between those of the positive and negative controls [24], [25]. The resistance of cells expressing the antibiotic resistance gene with no Tat signal sequence (V_H_3-BLA [-], negative control) was lower than that of cells expressing any other construct, with the notable exception of the V_H_2 (HuCAL) construct (ssTorA-V_H_2-BLA). Cells expressing V_H_2 exhibited the lowest ampicillin resistance, even lower than that of the negative control. Since most of the V_H_2 was expressed exclusively as inclusion bodies (Figure 3A), the biostatic effect of V_H_2 aggregate formation in *E. coli* might have further slowed cell growth beyond the bactericidal effect of the antibiotic.

### Screening of human germ-line V_H_ library via TAPE

In the Tat-associated screening system using ampicillin-containing agar plates, false-positive clones containing small V_H_ peptide fragments were often enriched because such fragments are highly compatible with the Tat pathway. To overcome this problem, previous screens have included a step to exclude clones with excessively high antibiotic resistance (i.e., counter-selection) [20]. In this study, to perform V_H_ solubility screening in a high-throughput manner, we enriched antibiotic-resistant clones in liquid cultures (‘liquid screen’) containing various concentrations of ampicillin (50–500 µg/ml) (Figure 2). Furthermore, to avoid enrichment of short V_H_ gene fragments that might yield false-positive results, full-size V_H_-BLA fusion genes were recovered by gel purification. In contrast to the limitation of library size in the plate-based method, the liquid screen with a culture larger than 100 ml can cover library sizes greater than 10^9^ because 1 ml overnight culture of *E. coli* in the LB with ampicillin contains normally about 10^9^ cells. The size of the human germ-line V_H_ library for TAPE was about 2.17×10^9^.

After the third round of TAPE through selection of antibiotic resistance, 154 V_H_ sequences were selected from the human germ-line V_H_ library that had been constructed using primers specific for the V_H_1, V_H_3, and V_H_5 families. These 154 V_H_ sequences were classified into 19 different V_H_ family types. Of the 154 total V_H_ hits, 146 (94.8%) were identified as members of the V_H_3 family; this frequency is significantly higher than the V_H_3 family frequency in the library prior to TAPE (101 V_H_3 family members out of 144 sequences: 70.1%). Among the V_H_3 family genes isolated from the germ-line V_H_ library, the V_H_3–30 and V_H_3–23 genes were predominant. On the other hand, the frequencies of the V_H_1 and V_H_5 families decreased by 0.1-fold and 0.3-fold, respectively. Overall, as a result of TAPE, the V_H_3 family was enriched 1.4-fold (i.e., from 70.1% to 94.8%), whereas the other families became less abundant (Table 1).

**Table 1:** Isolated germ-line V_H_ genes after the third round of TAPE.

V gene name	% of V_H_ after TAPE^a^	% of V_H_ before TAPE^b^	Foldincrease^c^
V_H_3-7	6.5	4.3	1.5
V_H_3-9	0.6	2.1	0.3
V_H_3-15	7.1	3.9	1.8
V_H_3-21	1.3	6.1	0.2
V_H_3-23	14.3	16.0	0.9
V_H_3-30	46.8	11.4	4.1
V_H_3-33	1.3	2.9	0.4
V_H_3-43	0.6	0.4	1.5
V_H_3-48	7.1	3.9	1.8
V_H_3-49	0.6	0.4	1.5
V_H_3-53	0.6	1.0	0.6
V_H_3-72	0.6	1.0	0.6
V_H_3-74	7.1	2.5	2.8
others	0	13.7	-
V_H_3 family	94.8	70.1	1.4
V_H_1-2	0.6	1.4	0.4
V_H_1-8	0.6	0.0	-
V_H_1-46	0.6	3.5	0.2
V_H_1-69	0.6	4.7	0.1
others	0	9.7	-
V_H_1 family	2.6	19.4	0.1
V_H_5-51	1.3	4.3	0.3
V_H_5-a	1.3	5.7	0.2
others	0	0.7	-
V_H_5 family	2.6	10.5	0.2

a Proportion of each identified V_H_ gene among the 154 sequences selected after TAPE.

b Proportion of each identified V_H_ gene among 144 sequences randomly selected from the library.

c Ratio of ‘% of V_H_ gene after TAPE’ to ‘% of V_H_ gene before TAPE’.

To determine the degree of soluble expression of isolated individual V_H_ domains lacking the BLA fusion, the soluble and insoluble fractions were separated after expression of the corresponding genes, and their expression patterns were compared with those of various V_H_ domains published previously [24], [25]. V_H_ domains randomly selected from the germ-line V_H_ library were expressed predominantly as inclusion bodies (Figure 3B, RD1–3), whereas the soluble expression levels of V_H_ domains selected by TAPE, e.g., MG4x4-44, MG4x4-25, MG10-10, and MG2x1, were significantly increased (Figure 3B). Moreover, the V_H_ domains selected by TAPE exhibited a higher ratio of soluble to insoluble protein than the previously characterized V_H_ domains described above, i.e., V_H_2 (HuCAL), V_H_3 (HuCAL), V_H_6 (HuCAL), V_H_3 (DP47d), and HEL4 (Figure 3A).

An artificial library comprising 25 individual V_H_ domains, either selected from the germ-line library or previously characterized V_H_ domains (HEL4, DP47d, HuCal V_H_3, and HuCal V_H_2) were subjected to TAPE. Only one clone, MG2x1, grew out at the third round of TAPE. This clone was used as the backbone for the frame-mutation library with selected mutation sites, described below.

### Screening of the frame-mutation library of MG2x1 via TAPE

**Figure 4:**
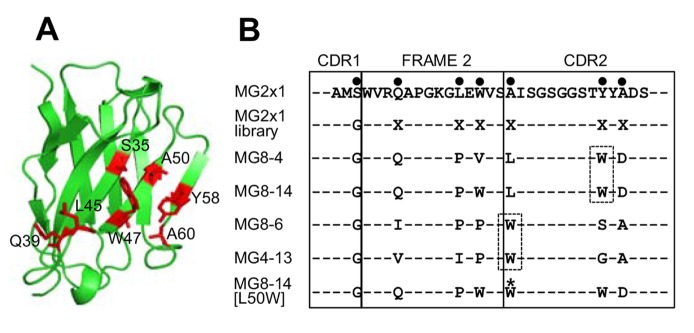
Rationale for designing of the combinatorial frame-mutation library. (A) Positions chosen for randomization based on the crystal structure of MG2x1. Residues are numbered according to the Kabat scheme for the V_H_ sequence. (B) Representative sequences (MG8-4, MG8-14, MG4-13, and MG8-6) selected from the MG2x1 frame-mutation library by TAPE were aligned with the original MG2x1 sequence. Mutation sites in the sequence of MG2x1 are shown as bold dots. All mutations were introduced using degenerate codons (NNK), except that serine (S) 35 was replaced by glycine (G). X represents all amino acids. At positions 50 and 58, the mutations converged primarily onto tryptophan, indicated by dashed boxes.

To confer additive solubility and stability to MG2x1, combinatorial mutations were introduced into seven specific sites of MG2x1 to generate the MG2x1 frame-mutation library. The number of distinct clones in the library was 1.4x10^8^, which covers all the possible combinations of mutations with NNK degeneration codon (theoretically, 6.4×10^7^ combinations). The selected mutation sites are distributed over the CDRH1 (S35), frame 2 (Q39, L45, and W47), and the CDRH2 (A50, Y58, and A60) with the kabat numbering system (Figure 4A, residues in red). These sites were selected by referring to the crystal structure of MG2x1 (PDB ID: 3ZHK) to identify amino acids that stretch their side chains outward from the surface. Also, all of these sites are located in the β-sheet structure away from the flexible loop of the CDRs. The frame-mutation library of MG2x1 was screened by TAPE, with the concentration of ampicillin increased (50, 100, 250, and 500 µg/ml) in successive rounds. After the final round of TAPE, 41 clones were randomly selected for sequencing of their V_H_ domains. Changes at positions 50 and 58 (Kabat scheme) were biased toward tryptophan (W): alanine (A) at position 50 was replaced by W in 39% (16/41) of the clones, and tyrosine (Y) at position 58 was replaced by W in 58% (24/41) of the clones (Table 2). The other mutation sites were not particularly biased. Sequence alignment of the selected V_H_ domains after TAPE revealed the biased amino acids at positions 50 and 58 (Figure 4B, dashed box). Based on the biased mutation frequencies at positions 50 and 58, we generated a MG8-14 mutant in which leucine (L) at position 50 was replaced with W (MG8-14 [L50W]) for further analyses of its physicochemical properties.

**Table 2:** Frequency of mutations of the randomly chosen V_H_ domains after screening of the combinatorial frame-mutation library of MG2x1 via TAPE.

Mutations	Q39	L45	W47	A50	Y58	A60	Codon Degeneracy (%)^a^
F	4		5	3	3		3.13
W	2	1	5	**16**	**24**		3.13
Y	3	5	1	2	3		3.13
A	2	2		3	1	7	6.25
C	2	1	2	5		7	3.13
I	4	4	4				3.13
L	3	7	3	6	3	1	9.38
M	3	1	6	2		1	3.13
P		6	4				6.25
V	5	5	6	3	1	1	6.25
G					1	1	6.25
N	3	1				4	3.13
Q	3						3.13
S	1	1		1	1	6	9.38
T	3	4	2		1	3	6.25
H			2			2	3.13
K	2	1					3.13
R	1	2	1		2	4	9.38
D						4	3.13
E					1		3.13
Total	41	41	41	41	41	41	

a Possible substitution of amino acids into the frame-mutation library (%) according to the given codon degeneracy of primers used for mutagenesis of MG2x1 as shown in the Table S1.

### Soluble expression level and thermodynamic stability are correlated in V_H_ domains selected by TAPE

**Figure 5:**
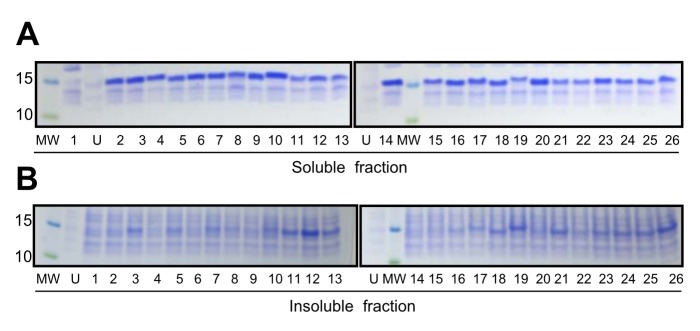
Soluble expression level of screened V_H_ domains. SDS-PAGE of soluble (A) and insoluble (B) fractions of *E. coli* expressing each V_H_ domains selected from the frame-mutation library of MG2x1 were loaded as follows: 1, V_HH_ (camel single-domain antibody); 2, HEL4; 3, MG2x1; 4, MG8-14; 5, MG2-47; 6, MG2-55; 7, MG2-57; 8, MG2-59; 9, MG4-2; 10, MG4-5; 11, MG4-6; 12, MG4-7; 13, MG4-12; 14, MG4-13; 15, MG4-17; 16, MG4-20; 17, MG4-28; 18, MG4-32; 19, MG4-33; 20, MG8-4; 21, MG8-5; 22, MG8-6; 23, MG8-8; 24, MG8-11; 25, MG8-12; 26, MG8-13. Lanes labeled ‘MW’ contained protein size markers (10 and 15 kD). U indicates fractions from a culture with no isopropylβD-1-thiogalactopyranoside.

**Figure 6:**
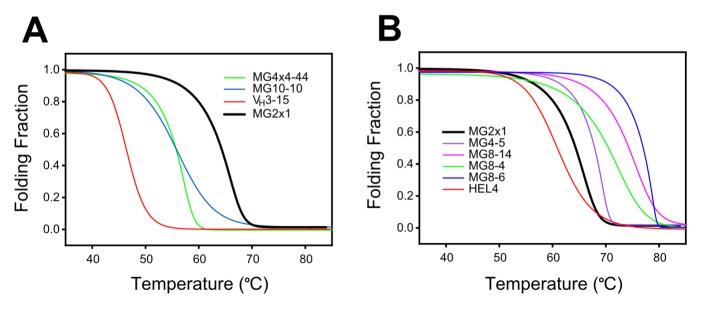
Thermodynamic stability. (A) Representative V_H_ domains selected from the germ-line library. (B) Representative V_H_ domains selected from the MG2x1 frame-mutation library. The black bold line indicates the profile of the parental V_H_, MG2x1, prior to mutation. Folding fraction was converted from the temperature-scouting CD profile at a fixed wavelength (230 nm).

Among the hits obtained from the combinatorial frame-mutation library of MG2x1, 23 unique sequences were selected from the final round of TAPE. Most of the selected V_H_ domains were expressed as soluble proteins. In particular, MG8-14, MG2-55, MG4-5, MG-4-13, MG8-4, and MG8-6 were expressed exclusively in their soluble forms (Figure 5). A previous study using the Tat pathway to express a protein fused to an antibiotic resistance marker showed that the ability to confer growth was correlated to both the solubility profile and the molecular weight of the protein [26]. The thermodynamic stabilities of the V_H_ domains selected from the naïve human V_H_ library by TAPE were higher than those of wild-type V_H_3 domains. The melting temperatures (T_m_) of the selected germ-line V_H_ domains were 55.6–65.2°C, whereas the T_m_ of the randomly chosen V_H_ domains from the germ-line library were generally below 50°C, e.g., 46.5°C for V_H_3–15 (Figure 6A). Among the selected germ-line V_H_ domains, MG2x1 had the highest T_m_. Furthermore, the T_m_ of V_H_ domains selected from the combinatorial frame-mutation library of MG2x1 (65.2–77.5°C) were significantly higher than that of the parental V_H_ (MG2x1) (Figure 6B). The thermodynamic stabilities of the engineered V_H_ domains identified in this study were generally higher than that of HEL4, which was selected from a combinatorial CDR3 library based on Dp47d by heat-resistant phage display selection [24].

### Selected V_H_ domains fold autonomously after denaturation

**Figure 7:**
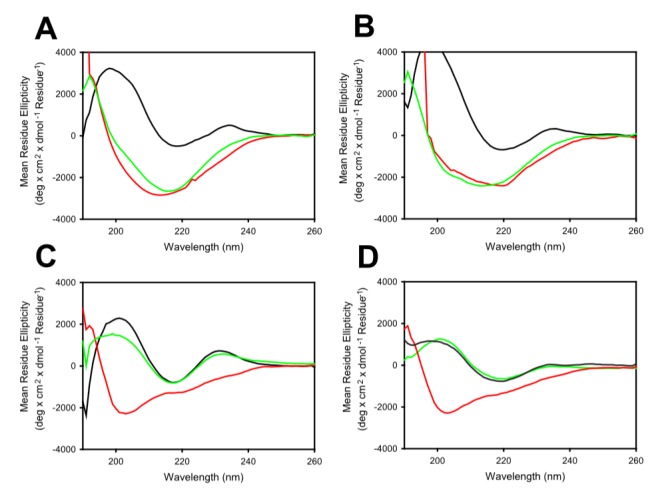
Far-UV CD spectra for the detection of reversible folding. (A) MG2x1. (B) MG8-6. (C) MG8-14. (D) Modified MG8-14 [L50W]. Black lines indicate profiles for V_H_ in native state at 25°C; red lines indicate the profile for V_H_ denatured at 85°C; and green lines indicate profiles for V_H_ renatured at 25°C.

Proteins exist in thermodynamic equilibrium between their folded and unfolded states. Hence, unstable proteins are much more vulnerable to heat and pH disturbance because exposure of their hydrophobic core during occupancy of the unfolded state promotes aggregation. Many V_H_3 family domains are soluble and aggregation-resistant. However, once these proteins are denatured, they never refold into their native conformation. This was the case for all V_H_ domains selected from the germ-line library in this study, including MG2x1. However, some of the V_H_ domains selected from the frame-mutation library of MG2x1 by TAPE were folded reversibly after denaturation. Far-UV circular dichroism (CD) spectra suggested that MG8-14 could be reversibly folded after denaturation heating at 85°C (Figure 7C), whereas the parental V_H_ domain, MG2x1, could not (Figure 7A). Furthermore, the modified MG8-14 [L50W] had a perfect renaturation profile (Figure 7D). MG8-6 had the highest T_m_, but could not refold after denaturation (Figure 7B). The recovery yield for the selected V_H_ after heat denaturation reached 95% (Table 3), in contrast to that of the parental sequence (MG2x1), which was below 5%.

**Table 3:** The recovery yields of selected V_H_ after thermal stress. Data are means and standard deviation for three independent treatment of heat denaturation within the same sample.

	Initial A_280 nm_ a	Final A_280 nm_ b	Recovery yield (%)^c^
MG2x1 (parent)	0.964±0.011	0.041±0.006	3.843±0.662
MG8-4	0.714±0.013	0.653±0.049	91.389±5.241
MG8-14	0.726±0.010	0.643±0.062	88.458±7.370
MG8-14 [L50W]	0.722±0.011	0.695±0.022	96.257±2.305
HEL4	0.964±0.011	0.878±0.032	91.097±2.348

a Absorbance at 280 nm at 25°C.

b Absorbance at 280 nm after heating (85°C) followed by cooling (25°C). Aggregates after heating were removed by centrifugation.

c The recovery yield was defined as the fraction of soluble V_H_ remaining after heating at the denaturation temperature (85°C).

### Structural features underlying the superior biophysical properties of selected V_H_ domains

**Figure 8:**
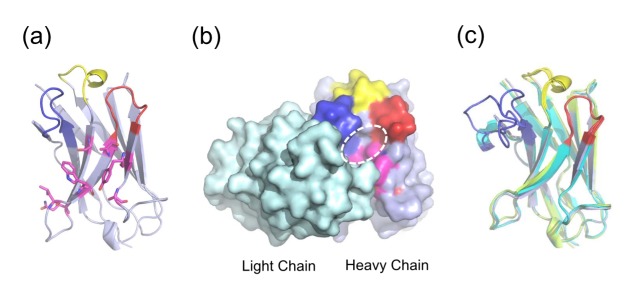
Structures of MG2x1, MG8-4, and MG8-14. (A) Structure of MG2x1 with CDRH1 (yellow), CDRH2 (red), and CDRH3 (blue). Mutation sites for the MG2x1 mutation library are indicated as sticks (magenta). (B) Antibody light chain, in surface rendering (cyan), is shown to highlight the relative locations of CDR H1–H3 and the mutation sites in MG2x1 (magenta). The circle indicates the cavity area. (C) Superposition of three V_H_ domains (MG2x1, MG8-4, and MG8-14), showing the variation in the loop in the CDRH3 region (blue).

Superimposition of crystal structures of the parental V_H_, MG2x1 (PDB ID: 3ZHK), and the modified V_H_ domains MG8-4 (PDB ID: 3ZHD) and MG8-14 (PDB ID: 3ZHL) revealed that these proteins have the same overall topology: two β-sheets connected by a disulfide bond between C22 and C96, yielding a typical β-sandwich lectin fold structure (Figure 8A and Table S2). The random amino acid changes introduced in the combinatorial frame-mutation library of MG2x1 are positioned on the β-strand that forms the sandwich scaffolds; in particular, they are located on the side of the sandwich corresponding to the hydrophobic interface region between heavy and light chains in the typical Fab complex arrangement (Figure 8B). Mutations in MG8-4 and MG8-14 altered the conformation of the flexible CDRH3 loop, whereas the CDRH1 and CDRH2 loops remained in their original conformations (Figure 8C and Table S2).

**Figure 9:**
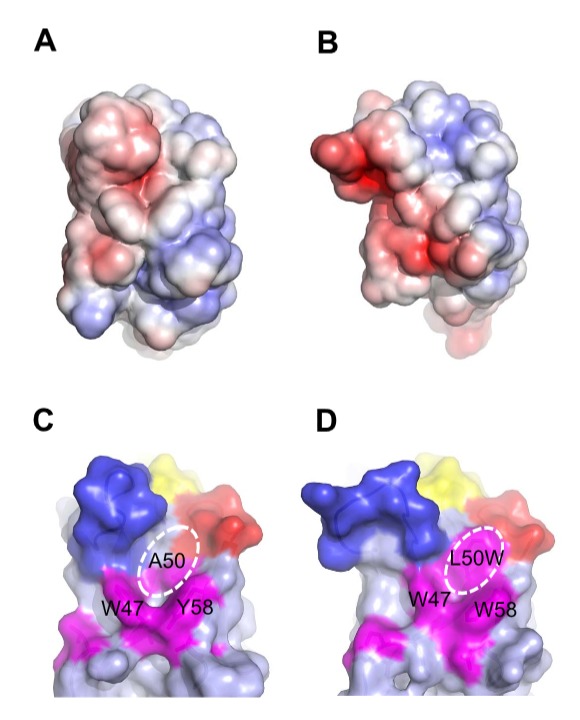
Surface features of MG2x1, MG8-14, and modified MG8-14 [L50W]. (A) Electrostatic charge distribution on the solvent-accessible surface of MG2x1 (red, −5 k; blue, +5 k). (B) Electrostatic charge distribution on the solvent-accessible surface of MG8-14. (C) Surface representation of MG2x1 showing the prominent cavity around residues 50 and 58. CDR regions are colored in yellow (CDRH1), red (CDRH2), and blue (CDRH3). Mutation sites are colored in magenta to highlight the cavity. (D) Surface representation of the structural model of the modified MG8-14 [L50W], in which L50 is replaced by W.

Surface electrostatic calculations revealed that MG8-4 and MG8-14 exhibited increased partial negative charge next to the hydrophobic patch, possibly due to the introduction of a charged group such as aspartate (D) at position 60, whereas substantial positive charge was detected next to the exposed surface of the heavy chain in all three structures (MG2x1, MG8-4, and MG8-14) (Figure 9A and 9B). The solvation energies of MG8-4 and MG8-14 (−1166.8 kcal/mol and −1153.4 kcal/mol, respectively) were significantly lower than that of MG2x1 (−1047.5 kcal/mol), suggesting that the charged residues on the surface contribute to the solvation energy, and hence the solubility, of the protein. Analysis of surface features revealed an significantly increased number of hydrogen bonds between side chains of the residues of MG8-4 and MG8-14 (26 and 39, respectively), whereas only 19 hydrogen bonds were observed in MG2x1, indicating that the architecture of MG8-14 is more stable than that of MG2x1. In addition, the structures of MG8-4 and MG8-14 contained more charge-charge interactions (8 and 9, respectively) than the structure of MG2x1 (5) (Table 4).

**Table 4:** Analyses of the structural features for MG2x1, MG8-4, and MG8-14.

	MG2x1	MG8-4	MG8-14	MG8-14 [L50W]
Solvent accessible surface (Å^2^)	5958.6	6009.2	6393.5	6141.6
Aromatic aromatic interaction	4	4	4	4
Main chain-sidechain hydrogen bonds	48	49	48	48
Side chain-side chain hydrogen bonds	19	26	39	35
Ionic interactions	5	9	8	8
Solvation energy (kcal/mol)	−1047.5	−1166.8	−1153.4	−1155.9

MG2x1 contains a prominent pocket comprising residues W47, A50, and Y58, with a cavity area of 32 Å^2^ and a volume of 19.5 Å^3^, centered at residue A50 (Figure 8B and Figure 9C). Sequence analysis of V_H_ domains selected by TAPE revealed that two positions in the framework, A50 and Y58, were consistently biased toward W. Residue A50 was also replaced by leucine (L) or W in representative selected V_H_ domains such as MG8-4, MG8-14, MG8-6, and MG4-13, suggesting that replacement of this residue with a bulky side chain is related to the stability of the molecule. The structural model of the modified MG8-14 [L50W] suggests that the cavity is filled with a triad bulky side chains consisting of 50 W, W47, and W58 (Figure 9D). Accordingly, the modified MG8-14 [L50W] exhibited high thermodynamic stability as well as reversible folding after heat denaturation (Figure 7D).

### Validation of the combinatorial CDRH synthetic library built on MG8-14 scaffold

**Figure 10:**
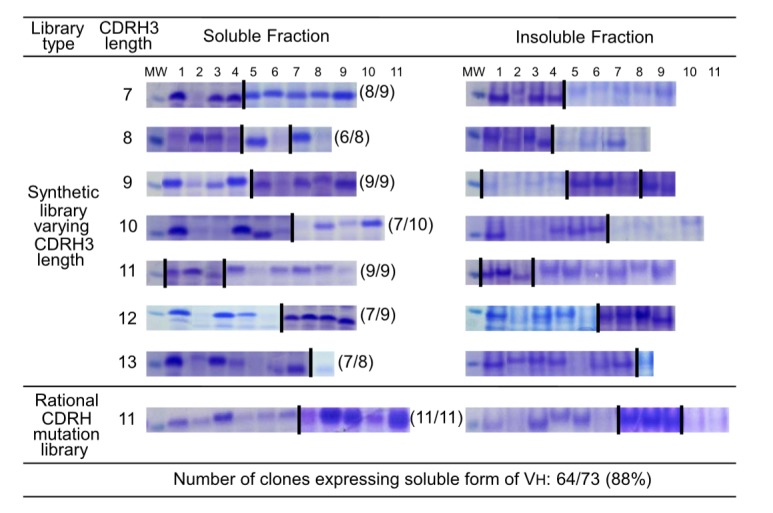
Validation of the combinatorial CDRH synthetic library built on MG8-14 scaffold. SDS-PAGE of soluble and insoluble fractions of *E. coli* expressing V_H_ domains selected randomly from the combinatorial CDRH3 synthetic libraries. Coomassie-stained gels are aligned by lane numbers (columns) and amino acid lengths of CDRH3 (rows). Images depict the region of the gel corresponding to the size of V_H_. Some images were combined with separate gels for the purpose of alignment (indicating with a dividing bar between gels). ‘MW’ indicates the protein size marker corresponding to a molecular weight of 15 kD.

To confirm the effects of CDR variation on the stability of V_H_ scaffold, we examined the soluble expression level of V_H_ domains containing CDRH3 regions of various lengths (7–13 amino acids), using a combinatorial CDRH synthetic library based on MG8-14. Eight or nine different sequences of each length were randomly selected and expressed in *E. coli*; 64 of 73 (88%) V_H_ clones were expressed in soluble form. In addition, 11 different sequences from a rational mutation library (CDRH3 length fixed and seven positions of CDRH1, 2 and 3 of MG8-14 were randomized) were randomly tested; all of the test sequences were expressed in soluble form in the cytoplasm of *E. coli* under reducing conditions (Figure 10). Thus, aggregation was infrequently occurred regardless of CDR alteration in a combinatorial CDRH library that used the MG8-14 framework as a scaffold.

### Humoral response to MG2x1 and MG8-14 in mouse

**Figure 11:**
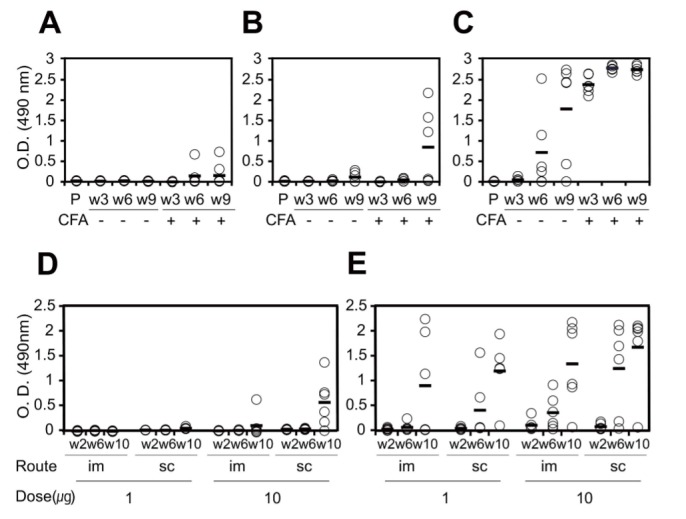
Humoral immune response of BALB/c mice. Six mice (per group) were received multiple intravenous injections of PBS (P), MG2x1 (A), MG2x1 plus CFA (A), MG8-14 (B), MG8-14 plus CFA (B), V_HH_ (C), or V_HH_ plus CFA (C) at week 3 (w3), week 6 (w6), and week 9 (w9). Six mice (per group) were received multiple injections of 1 µg MG8-14 intramuscularly (im, D), 1 µg MG8-14 subcutaneously (sc, D), 10 µg MG8-14 intramuscularly (im, D), 10 µg MG8-14 subcutaneously (sc, D), 1 µg V_HH_ intramuscularly (im, E), 1 µg V_HH_ subcutaneously (sc, E), 10 µg V_HH_ intramuscularly (im, E), or 10 µg V_HH_ subcutaneously (sc, E) at week 2 (w2), week 6 (w6), and week 10 (w10).

To test the humoral immune response of the selected VH domains, BALB/c mice were subjected to repeated immunization with selected V_H_ domains, administered by various routes. Antibody against MG2x1 was undetectable after nine intravenous injections of 10 µg protein over 9 weeks (Figure 11A). Furthermore, there was no antibody-boosting response, even when injections included Freund′s Complete Adjuvant (CFA), in four of six mice at week 9 (Figure 11A). In the case of MG8-14, there was no detectable anti–MG8-14 antibody until week 6, although a mild antibody response was present in half of the tested mice at week 9 (Figure 11A). On the other hand, a camel single-domain antibody, V_HH_ [23], was more immunogenic than MG2x1 and MG8-14, as shown by the high titer after the first injection (with CFA) at week 3 (Figure 11B). Intramuscular and subcutaneous injection of 1 µg MG8-14 resulted in no antibody response against MG8-14 throughout a 10-week course of immunization (Figure 11D), whereas V_HH_ injection caused an increase in antibody titer starting at week 6 (Figure 11E). When mice were injected intramuscularly with 10 µg MG8-14, anti-MG8-14 antibody was elicited moderately at week 10 in only one of six mice. Subcutaneous injection of 10 µg MG8-14 elicited no antibody response until the fourth injection at week 6; moderate levels of anti-MG8-14 antibody were detectable after this time point (Figure 11D). Among mice subjected to intramuscular and subcutaneous injection of V_HH_, most animals exhibited an anti-V_HH_ antibody response at week 4, immediately after the second injection (Figure E).

## Discussion

The external diameter of the TatABC complex is around 160 Å, but its pore is relatively small [27]. Variations in complex size may result in variations in pore size, influencing the compatibility of each complex with differently sized Tat substrate proteins [28]. The capacity of the Tat system to export proteins via membrane-bound TatABC complexes varies among species of Gram-negative bacteria. For example, the *A. tumefaciens* TatABC complex is capable of exporting large (>80 kD) proteins [29], whereas in *E. coli*, the correlation between protein folding and export to the periplasm via the Tat pathway is poorer for proteins larger than 30 kDa than proteins of a lower molecular weight [26]. The molecular weight of the V_H_ domain is around 14 kDa; therefore, this group of proteins was predicted to be compatible with the Tat pathway of *E. coli*. Consistent with this expectation, in this study, the export of V_H_ *in vivo* corresponded well with properties related to protein stability *in vitro*. Accordingly, because the V_H_3 family is the most soluble of the seven V_H_ families (V_H_1–7), the V_H_3 family was enriched via TAPE (Table 1) in a screen of a human germ-line library. This suggests that selection was driven by the function of the Tat pathway, which serves as a ‘molecular sieve’ *in vivo* as already discussed in many previous works [28], [30], [31].

We tried to compare ampicillin resistance of V_H_ variants to the other variants by using visual measurement. For example, spot analyses of serial diluents of the culture containing ampicillin [22] was not sensitive to demonstrate the direct comparison of their resistance in this study (data not shown). To overcome this limitation, we performed a head-to-head competition of the ampicilline resistance among the 25 germ-line V_H_ domains (the artificial library) with the third round of selection in liquid culture. This experiment resulted in MG2x1 as a sole survivor, a V_H_3 family member (V_H_3–23), which was used for the backbone of a frame-mutation library. This library was then subjected to another round of TAPE, with the goal of improving the physicochemical properties of this protein. Considering that MG2x1 is already relatively soluble and stable, one might expect only a marginal improvement from directed evolution via TAPE. However, subjecting the frame-mutation library to selection resulted in a significant improvement in folding-related properties.

Studies of the protein folding quality control mechanism of the *E. coli* Tat pathway have primarily focused on the tendency of proteins to be expressed in soluble form [19], [20]. However, the correlation between the selection via Tat-mediated protein folding and increases in the thermodynamic stabilities of proteins of interest has not been clearly demonstrated. In this study, we showed that both protein expression in soluble form and properties related to thermodynamic stability were clearly improved by Tat-associated screening. Foit et al. also demonstrated that antibiotic resistance bestowed by the tripartite fusion protein is correlated with stability *in vivo* and thermodynamic stability *in vitro* [32]. Although both methods use the same reporter gene, i.e., *BLA*, the protein folding occurs in a different environment, i.e., periplasm for the tripartite system and cytoplasm for TAPE. With the reduced condition of TAPE for protein folding, some of the evolved V_H_ was capable of autonomous refolding over repeated cycles of heating and cooling. More reversible refolding and a higher recovery yield should increase resistance to mechanical or thermal stresses during the purification process, as well as improve long-term storage due to the low exposure rate of hydrophobic patches [33].

Christ et al. demonstrated that the frequency of aggregation-resistant domain was about 80% in the repertoire after heat-cooling selection and about 71% in the large aggregation-resistant repertoire generated by combinatorial ligation of CDR-encoding regions [34]. In this study, the frequency of aggregation-resistant V_H_ domains in combinatorial CDRH3 repertoires with a fixed scaffold (MG8-14) screened by TAPE was 88%, regardless of the length of the CDRH3 region (Figure 10). With the exception of the CDRH3 region, the crystal structure of MG8-4 and MG8-14 superimposed closely with the parental V_H_, MG2x1, despite containing mutations in the frame region (Figure 8C). In addition, the atomic mobility of MG8-14 at residue L50 had the lowest observed B-factor (32), whereas the average B-factor was 43.3. These observations suggest that the core of this region is very rigid, but is still capable of accommodating various structures of CDRH3. As framework and CDR regions of the scaffold are conformational, a stability-functional tradeoffs are fully anticipated when the stability-enhancing mutation are introduced to the given functional protein, for example, scFv [20]. In contrast, we screened out the stable V_H_ scaffold first and then generated the combinatorial CDRH synthetic library to give functionality later. As the affinity of V_H_ domains we screened from the library against several antigens, including HER3, TNF-α, and albumin were all sub-nanomolar range, we can expect that the problems on a stability-functional tradeoffs would be a minimal when we screen the functional V_H_ domains with this quality of the library (data not shown).

The modified MG8-14 [L50W] contains three W residues that fill a large cavity of MG2x1 near the V_H_/V_L_ interface. Van der Waals interactions in this region would enhance stable architecture, allowing reversible folding of the antibody during the refolding process after denaturation. Within the cavity structure, high temperature leads to thermal destabilization as a result of water permeation [35], [36]. Therefore, water molecules in the hydrophobic cavity of MG2x1 may directly affect thermal resilience and promote structural perturbation. Taken together, these data demonstrate that surface properties are important factors in selection of single-domain antibodies with high solubility and thermodynamic stability.

V_H_ domains that had been selected by heat-denatured phage display from a combinatorial CDR repertoire exhibited an enrichment of certain amino acids at several positions within the CDR regions, including glycine at position 35 and glutamate at position 32 [37]. Our differentiated *in vivo* selection strategy, using the Tat pathway in *E. coli*, resulted in a unique preference for tryptophan at positions 50 and 58, leading to the creation of a bulky ring structure. We believe that this preference helps V_H_ to acquire a stable conformation, preventing structural perturbation during folding and refolding.

MG2x1 contains a negatively charged amino acid, aspartic acid (D) at position 61, which was previously identified as a determinant of protein aggregation and solubility [38]. In MG8-4 and MG8-14, which were selected from the MG2x1 frame-mutation library, D was incorporated consecutively at positions 60 and 61, significantly increasing the net negative charge. This preference for adjacent D residues has also been observed in other protein stability screens of combinatorial CDR repertoires. For example, positions 32 and 33 of V_H_ and positions 52 and 53 of V_L_ are determinants for aggregation resistance [37].

One important safety issue in protein therapeutics is related to immunogenicity. Many previous studies suggest that formation of sub-visible aggregates exerts a major influence on the humoral immune response [39], [40]. In this work, the antibody titer represents both the quantity and quality (affinity) of IgG that is specific to certain V_H_ domain. Although we cannot discriminate which factor affects the titer more than the other does, it is obvious that the mouse immune system hardly responded to the selected V_H_ domains even with CFA, compared to V_HH_ as shown in Figure 11. This may be attributed to a favorable folding properties of the selected V_H_ domains preventing aggregation, as we employed Tat-associated protein folding fitness filter.

### Database access codes

The atomic coordinates and structure factors have been deposited in the Protein Data Bank. www.pdb.org (PDB ID: **3ZHL**, **3ZHK** and **3ZHD**).

## Supporting Information

Figure S1 **SDS-PAGE of the purified V_H_ domains used for the measurement of Far-UV CD spectra.** (A) V_H_ domains for testing thermodynamic stability were loaded as follows: 1, MG4x4-44; 2, MG10-10; 3, MG3-15; 4, MG2x1; 5, HEL4; 6, MG4-5; 7, MG8-14; 8, MG8-4; 9, MG8-6. (B) V_H_ domains for testing reversible folding were loaded as follows: 1, MG2x1; 2, MG8-6; 3, MG8-14; 4, modified MG8-14 [L50W]. Lanes labeled ‘MW’ contained protein size markers.
(PDF)

Table S1 **Oligonucleotides used in this study.**
(PDF)

Table S2 **Data collection and structure Solution parameters.**
(PDF)
