# Correction: Directed Evolution of Human Heavy Chain Variable Domain (V_H_) Using *In Vivo* Protein Fitness Filter

**DOI:** 10.1371/journal.pone.0102631

**Published:** 2014-07-07

**Authors:** The PLOS ONE Staff

## Notice of Republication

This article was republished on June 16, 2014, due to a conversion error resulting in illegible figures in the online version. The publisher apologizes for these errors. Please see this article again to view the corrected figures.
