# Supplementary figures and images for: Directed Evolution of Human Heavy Chain Variable Domain (VH) Using In Vivo Protein Fitness Filter
Source: PLoS One. 2014 Jun 3;9(6):e98178. doi: 10.1371/journal.pone.0098178 (PMC4043505; doi:10.1371/journal.pone.0098178)

Supporting Figure S1

**A**

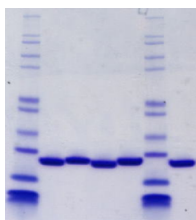

MW

1

2

3

4

MW

5

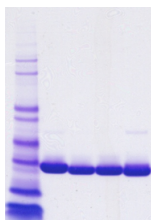

MW

6

7

8

9

**B**

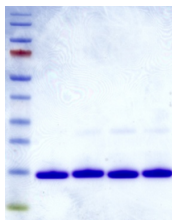

MW

1

2

3

4

Supplement: Figure S1 — SDS-PAGE of the purified VH domains used for the measurement of Far-UV CD spectra. (A) VH domains for testing thermodynamic stability were loaded as follows: 1, MG4x4-44; 2, MG10-10; 3, MG3-15; 4, MG2x1; 5, HEL4; 6, MG4-5; 7, MG8-14; 8, MG8-4; 9, MG8-6. (B) VH domains for testing reversible folding were loaded as follows: 1, MG2x1; 2, MG8-6; 3, MG8-14; 4, modified MG8-14 [L50W]. Lanes labeled ‘MW’ contained protein size markers. (PDF) [file pone.0098178.s001.pdf]
