# Supplementary material for: Directed Evolution of Human Heavy Chain Variable Domain (VH) Using In Vivo Protein Fitness Filter
Source: PLoS One. 2014 Jun 3;9(6):e98178. doi: 10.1371/journal.pone.0098178 (PMC4043505; doi:10.1371/journal.pone.0098178)
Supplement: Table S1 — Oligonucleotides used in this study. (PDF) [file pone.0098178.s002.pdf]

Supporting Table S1. Oligonucleotides used in this study.

Number	Primer sequences[a]	Note[b]
1	GCCATATGAACAATAACGATCTCTTTCAGG CATCACGT	
2	GCGGATCCATGGTGGTGATGGTGGTGTGC GGCCGCTGAAGAGACGGTCACCAACGTGCC	
3	GCGCGGCCGCACACCCAGAAACGCTGGTG	
4	GCGGATCCTTACCAATGCTTAATCAGTGAGGC	
5	GCCCATGGCAGGTKCAGCTGGTGCAG	VH1a, sense
6	GCCCATGGCAGGTCCAGCTTGTGCAG	VH1b, sense
7	GCCCATGGSAGGTCCAGCTGGTACAG	VH1c, sense
8	GCCCATGGCARATGCAGCTGGTGCAG	VH1d, sense
9	GCCCATGGGARGTGCAGCTGGTGGAG	VH3a, sense
10	GCCCATGGCAGGTGCAGCTGGTGGAG	VH3b, sense
11	GCCCATGGGAGGTGCAGCTGTTGGAG	VH3c, sense
12	GCCCATGGGARGTGCAGCTGGTGCAG	VH5a, sense
13	GCGCGGCCGCTGAGGAGACGGTGAC	JH1,4,5,6, antisense
14	GCGCGGCCGCTGAGGAGACAGTGAC	JH2, antisense
15	GCGCGGCCGCTGAAGAGACGGTGAC	JH3, antisense
16	GCCCATGGGAAGTCCAACTGGTTGAATCTGGTG GCGGTTTAGTT	
17	AGTTGAACCGCCAGAGCCGGAAATMNNTGAGAC MNNTTCMNNACCTTTGCCTGGCGCMNNACGCAC CCAGCCCATAGCATAAGAAGAAAAGGTAAAGCCA CTTGCAGCACAGCT	
18	ATTTCCGGCTCTGGCGGTTCAACTNNKTACNNKG ATAGCGTTAAAGGTCGTTTCACAATCTCC	
19	GCGCGGCCGCACTGCTCACAGTAACCAGGGTAC CCTG	

[a]K means G or T; S means C or G; R means A or G; M means A or C; and N means A, T, G, or C

[b]Sense and antisense primers sets for amplifying the V_H1, V_H3, and V_H5 genes (http://vbase.mrc-cpe.cam.ac.uk)
