# Supplementary material for: Directed Evolution of Human Heavy Chain Variable Domain (VH) Using In Vivo Protein Fitness Filter
Source: PLoS One. 2014 Jun 3;9(6):e98178. doi: 10.1371/journal.pone.0098178 (PMC4043505; doi:10.1371/journal.pone.0098178)
Supplement: Table S2 — Data collection and structure Solution parameters. (PDF) [file pone.0098178.s003.pdf]

Supporting Table S2. Data collection and structure Solution parameters.

Crystal type	MG2x1	MG8-4	MG8-14
Unit cell parameters (Å)	a=104.41 b=55.70 c=74.53 σ=90 β=126.4 γ=90	a=87.07 b=87.07 c=55.73 σ=90 β=90 γ=120	a=56.20 b=56.20 c=81.52 σ=90 β=90 γ=120
Resolution (Å)	1.96 – 31.97	1.96 – 34.3	2.47 – 28.1
Space group	C 2	P3 2 1	$P3_2$ 2 1
Completeness (%)	99.2 (92.5)[a]	99.3 (86.7)[a]	99.9 (100)[a]
R_{sym} (%)[b]	9.9 (31.2)	11.3 (42.3)	10.3 (31.7)
I / σ (%)	39.1 (9.3)	48.4 (10.8)	53.9 (15.0)
No. of refined atoms Protein / water	1794 / 216	1783 / 281	919 / 44
R_{factor} / R_{free} (%)[c]	25.2 (29.7)	17.7 (23.1)	19.1 (24.5)
r.m.s.d bond length (Å)	0.009	0.008	0.006
r.m.s.d bond angle	1.1	1.2	1.1
Ramachandran plot (%)			
Most favored region	98.3	96.2	99.2
Additionally allowed region	1.7	3.8	0.8
Residues in outlier region	0	0	0

[a]Numbers in parentheses are statistics from the highest resolution shell.

[b]$R_{sym} = \Sigma|I_{obs} - I_{avg}|/ \Sigma I_{obs}$, where I_{obs} is the observed individual reflection and I_{avg} is the average over symmetry equivalents.

[c]$R_{factor} = \Sigma||F_0| - |F_c||/ \Sigma|F_0|$, where $|F_0|$ and $|F_c|$ are the observed and calculated structure factor amplitudes, respectively. R_{free} was calculated using 5% of the data.
